# Plasma Cytokine Profiling Reveals Differences between Silicotic Patients with Simple Silicosis and Those with Progressive Massive Fibrosis Caused by Engineered Stone

**DOI:** 10.3390/ijms24021541

**Published:** 2023-01-12

**Authors:** Antonio Campos-Caro, Gema Jiménez-Gómez, Alejandro García-Núñez, Antonio Hidalgo-Molina, Antonio León-Jiménez

**Affiliations:** 1 Biomedical Research and Innovation Institute of Cadiz (INiBICA), 11009 Cádiz, Spain; 2 Research Unit, Puerta del Mar University Hospital, 11009 Cádiz, Spain; 3 Genetics Area, Biomedicine, Biotechnology and Public Health Department, School of Marine and Environmental Sciences, University of Cádiz, 11510 Cádiz, Spain; 4 Pulmonology, Allergy and Thoracic Surgery Department, Puerta del Mar University Hospital, 11009 Cádiz, Spain

**Keywords:** engineered stone, artificial stone, quartz agglomerate, silicosis, cytokines, biomarkers, human

## Abstract

Engineered stone silicosis has become an occupational epidemic disease that progresses rapidly to progressive massive fibrosis with respiratory failure and death, and there is no effective treatment. Silica deposition in the lung triggers a series of inflammatory reactions with the participation of multiple cytokines and cellular mediators whose role in the development and progression of the disease is largely unknown. We hypothesized that differences in plasma cytokine levels exist between patients diagnosed with simple silicosis (SS) and patients diagnosed with progressive massive fibrosis (PMF). Plasma samples from 91 ES silicosis patients, diagnosed and classified by chest radiography and/or high-resolution computed tomography with SS (*n* = 53) and PMF (*n* = 38), were assayed by multiplex assays for levels of 34 cytokines. Additionally, a healthy volunteer control group (*n* = 22) was included. Plasma levels of a high number of cytokines were significantly higher in subjects with silicosis than in healthy control subjects. Moreover, the levels of IL-1RA, IL-8, IL-10, IL-16, IL-18, TNF-α, MIP-1α, G-CSF and VEGF were significantly elevated in PMF compared to SS patients. This study shows that plasma cytokine levels differ between healthy people and silicosis patients, and some of them are also significantly elevated in patients with PMF compared with patients with SS, which could indicate their involvement in the severity of the disease, be considered as biomarkers and could be explored as future therapeutic targets for the disease.

## 1. Introduction

Silicosis is a diffuse interstitial lung disease caused by the inhalation of free microscopic particles of crystalline silica and is one of the more common diseases related to work activities that can lead to incapacitating lung fibrosis and respiratory failure [1]. Traditionally related to mining or drilling activities, in recent years, the cases of this type of disease have been reduced in developed countries by the implementation of health and safety rules in workplaces. However, silicosis has re-emerged in the last 20 years as a consequence of the use of a new material, artificial stone (AS) or engineered stone (ES), mainly composed of crystalline silica and synthetic resins and frequently used for manufacturing kitchen and bathroom countertops; silicosis has also been considered an occupational epidemic in some countries [2,3,4,5,6,7]. ES silicosis is characterized by a short latency period and by a greater aggressiveness than classic silicosis [8,9], and the progression of this entity continues to produce progressive massive fibrosis (PMF) in up to 40% of patients even after four years of cessation of their exposure to silica [10]. 

Although the physiopathological origin of ES silicosis is not completely defined, as has occurred for traditional silicosis, it is thought to start with the interaction of silica crystals with immune cells, mainly macrophages but not alone, present in the alveoli [11,12]. Struggling to eliminate injury, activated cells engulf and try to degrade silica particles, but this is avoided due to the toxic nature of crystalline silica particles. Inflammasome activation initiates cytokine cascade signaling, resulting in a chronic inflammatory process and the development of lung fibrosis.

Cytokines are the main mediators of intercellular communication regulating a wide range of cellular processes, including activation, proliferation, differentiation, survival/apoptosis, inflammation, fibrosis, tissue repair and hematopoiesis, among others. Our previous studies suggest that ES silicosis patients maintain a systemic inflammatory condition even years after the cessation of exposure to silica dust [13]. To date, many groups have analyzed one or a few specific cytokines in serum, sputum, or bronchoalveolar lavages from groups of patients with silicosis due to different causes, but to date, no one has analyzed such a large and homogeneous group of patients or a broad panel of cytokines that may be involved in the development of silicosis caused by ES.

We hypothesized that patients with ES simple silicosis (SS) or with PMF, a more advanced stage of the disease, will exhibit differences in their systemic cytokine profiles. The progression rate of each patient could be a helpful tool for clinicians. Furthermore, the observed changes in systemic cytokine levels could reflect the intrinsic pathophysiological basis for directing new or existing treatments that could be used to slow or stop the progression of the disease. 

## 2. Results

### 2.1. Characteristics of the Study Population

A total of 91 patients with silicosis accepted participation in the study, of whom 53 were diagnosed with SS and 38 with PMF. All subjects studied were males, and their sociodemographic data are shown in Table 1. Mean age, starting age and duration of exposure to engineered stone dust were all similar, without significant differences between the groups studied. A healthy control (HC) group not exposed to silica dust was also studied. The participants were categorized by smoking status as follows: “non-smoker” (never smoker); “ex-smoker” (smoking cessation at least 1 year before blood draw); and “smoker” (current smoking at the time of blood draw). Since smoking may have some effects on inflammation and therefore likely to affect the cytokine profile in smoking subjects, we decided to check all the analyses after removing the few smoking subjects and we did not see any difference in the results from leaving them in the study. Therefore, we decided to consider and include all subjects in the study regardless of their smoking status.

### 2.2. Analysis of Plasma Cytokines

We decided to explore the potential association of circulant cytokine levels with ES silicosis disease, broadly accepted as a chronic inflammatory process leading to pulmonary fibrosis. The effects or functions of cytokines are highly pleiotropic and redundant in their functions, which makes their classification difficult, but we clustered the cytokines analyzed into five large groups, bearing in mind that some of them could be classified into different groups.

#### 2.2.1. Pro-Inflammatory Cytokines

**Figure 1:**
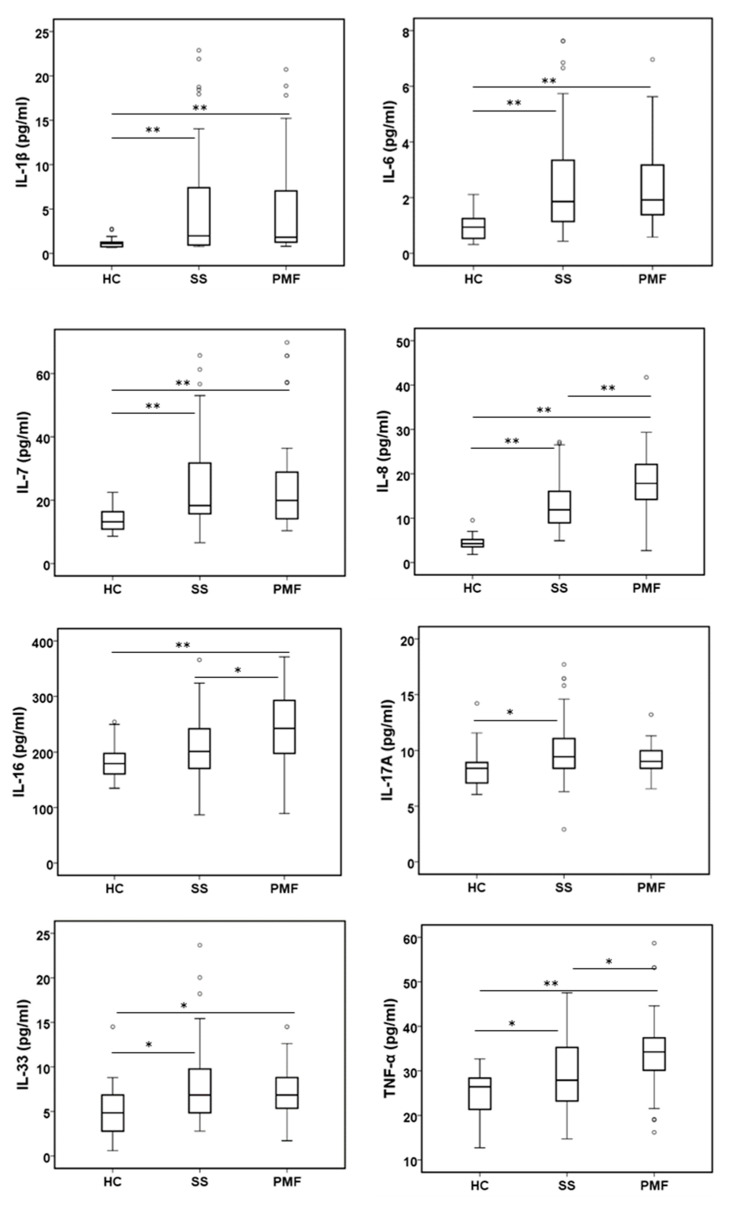
Levels of inflammatory cytokines in plasma from peripheral blood were assayed in patients diagnosed with simple silicosis (SS), progressive massive fibrosis (PMF) and healthy controls (HC). All cytokines were assayed by the Bio-Plex assay, except IL-16 and IL-33, which were assayed by R&D Systems Assay. * and ** indicate significance *p* < 0.05 and *p* < 0.01, respectively.

The plasma pro-inflammatory cytokine levels of the SS and PMF patient groups were, in general, elevated compared with those of the control group (Figure 1). The mean plasma levels of IL-1β, IL-6, IL-7, IL-8, IL-17A, IL-33 and TNF-α were significantly higher in the patient groups than in the control group. Although the IL-16 level followed the same trend, it was only significantly increased in the PMF group compared to the HC or SS groups. However, notable significant differences in the levels of the cytokines IL-8 (also considered a chemokine), IL-16 and TNF-α were observed between the SS and PMF patient groups.

#### 2.2.2. Anti-Inflammatory Cytokines

**Figure 2:**
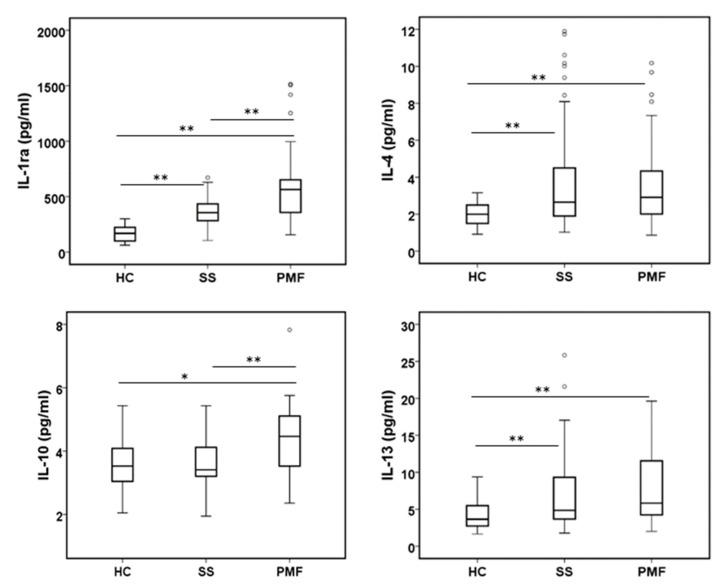
Levels of anti-inflammatory cytokines in plasma from peripheral blood were assayed in all participants. All cytokines were assayed by the Bio-Plex assay. * and ** indicate significance *p* < 0.05 and *p* < 0.01, respectively.

Analysis of the main anti-inflammatory cytokines also showed a general increase (Figure 2), but some differences were observed between group comparisons. Therefore, IL-1RA, IL-4 and IL-13 levels were higher in patients diagnosed with SS or PMF than in HCs. Furthermore, within groups of diagnosed patients with silicosis, IL-1RA and IL-10 levels were higher in PMF than in SS patients. However, the IL-10 level did not show differences between HCs and SS patients, but it did in the comparison of either of these groups with patients diagnosed with PMF.

#### 2.2.3. TH1/TH2 Cytokine Profiles in Diagnosed Silicosis Patients

**Figure 3:**
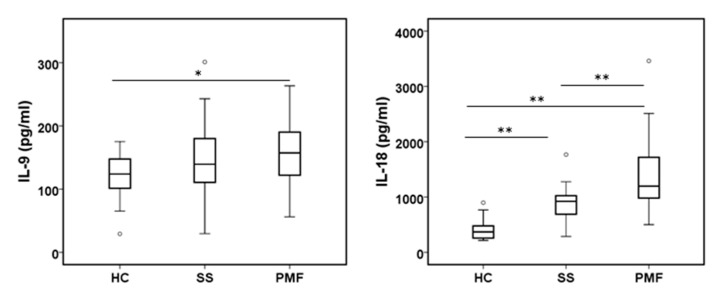
Levels of TH1/TH2 response cytokines in plasma from peripheral blood were assayed in all participants. IL-9 cytokine levels were assayed by the Bio-Plex assay, and IL-18 levels were assayed by the R&D Systems Assay. * and ** indicate significance *p* < 0.05 and *p* < 0.01, respectively.

As T lymphocytes have a key role in inflammatory processes, we analyzed the status of some cytokines implicated in the TH1 (INF-γ, IL-2, IL-12p70, IL-18) and TH2 (IL-3, IL-5, IL-9, IL-15, IL-23) responses. Plasma levels of IL-2, IL-12p70, IL-5 and IL-23 cytokines were all under the level of detection to be analyzed and compared. IL-15 levels were undetectable in the HC group (not shown), and for this reason, it was not further analyzed, but it was detected in the SS and PMF groups, suggesting that IL-15 was increased in ES silicosis patients. As shown in Figure 3, the main cytokines involved in the TH1/TH2 responses, i.e., INF-γ and IL-3, did not show significant differences between any of the groups studied. The exceptions were IL-4 and IL-13, considered cytokines related to the TH2 response, but we have previously shown them as anti-inflammatory cytokines (see Figure 2), and IL-9 levels were only significantly different between HC and PMF groups but not between HC and SS or between SS and PMF. A notable exception was IL-18 levels, which showed a progressive and highly significant increase in the direction HC → SS → PMF and are considered to facilitate both TH1/TH2 responses [14].

#### 2.2.4. Chemokines

**Figure 4:**
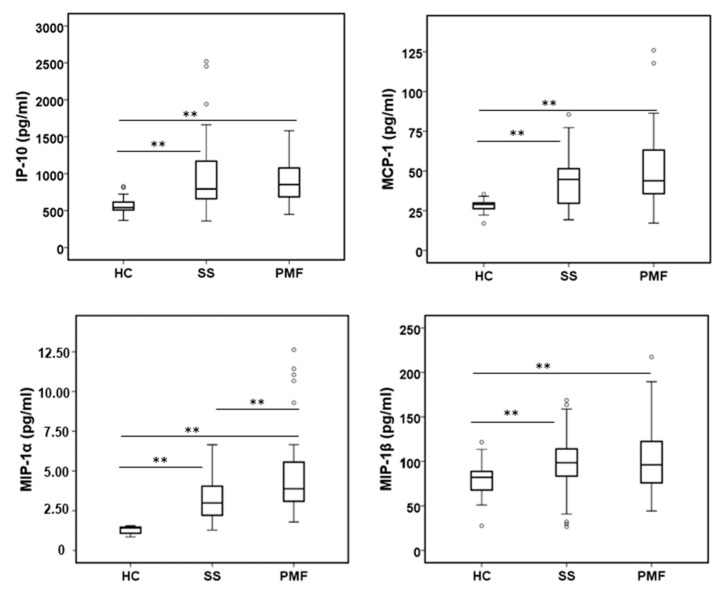
Levels of chemokines in plasma from peripheral blood were assayed in all participants. All chemokines were assayed by the Bio-Plex assay. ** indicate significance *p* < 0.01.

The levels of some chemokines, such as CXCL1/GRO-α, IP-10, MCP-1, RANTES, Eotaxin, MIP-1α and MIP-1β, were also measured. Among them, CXCL1/GRO-α, Eotaxin and RANTES did not show differences between the groups studied (not shown). Others, such as IP-10, MCP-1, MIP-1α and MIP-1β, showed significant differences when either of the silicotic groups was compared to the HC group, as shown in Figure 4. Additionally, MIP-1α levels showed a significant increase in PMF patients compared to SS patients. IL-8, also considered a strong chemokine, was previously analyzed in the pro-inflammatory cytokine group (see Figure 1).

#### 2.2.5. Growth Factors

**Figure 5:**
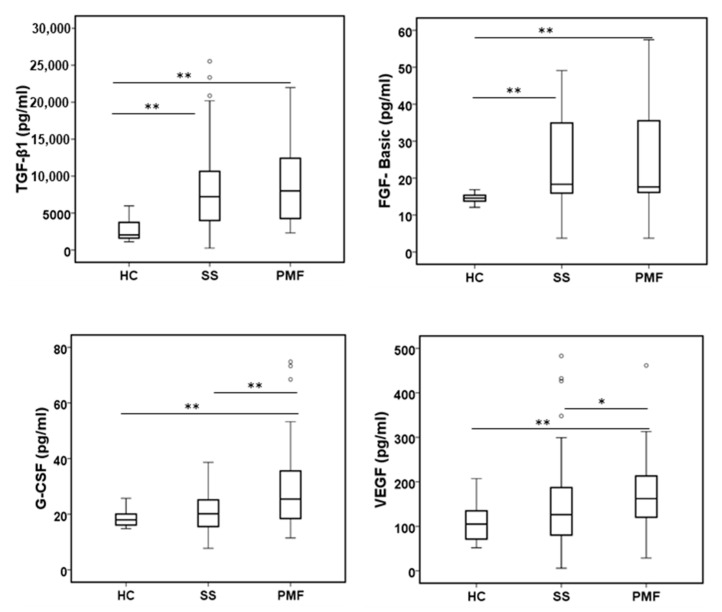
Levels of growth factors in plasma from peripheral blood were assayed in all participants. All growth factors were assayed by the Bio-Plex assay except TGF-β1, which was assayed by a Human TGF-β1 Elisa Kit from Sigma-Aldrich. * and ** indicate significance *p* < 0.05 and *p* < 0.01, respectively.

Finally, several growth factors were also analyzed, and the results are shown in Figure 5. TGF-β1, considered the main profibrotic factor, showed a significant increase in the SS and PMF groups compared to the HC group but not between the SS and PMF groups. A similar pattern to TGF-β1 was observed for basic FGF. In the case of VEGF and G-CSF, a weak increasing trend is observed from HC to SS, but it is only significant when we compare any of these groups with the PMF group. Other factors, such as PDGF-BB and GM-CSF, did not present differences at all between the studied groups (not shown).

As a summary, all the cytokine levels that are altered, and those that are not, among the different groups studied are included in Table 2.

## 3. Discussion

Previously, it has been shown that patients diagnosed with ES silicosis continue progressing to more advanced stages of the disease and present a chronic inflammatory process even years after exposure cessation [10,13]. Several groups have reported different plasma/serum cytokines and other molecules as biomarkers in silicosis caused by different agents from ES (miners, quarry workers, etc.) [15,16,17,18,19,20,21,22,23,24]. However, ES silicosis has some differences from silicosis originating from natural stones, characterized by faster radiological progression, a decline in lung function and mortality [9]. In addition, scarce data are available regarding plasma biomarkers, specifically in a numerous and well-studied group of patients with ES silicosis. Knowing which are the altered cytokines in ES silicosis patients could guide us for future treatments to stop or slow the progression of the disease. To our knowledge, this is the first study to analyze plasma cytokine levels in patients postexposure to ES, and furthermore, it uses a broad panel of cytokines, some of which have not been previously evaluated in patients with silicosis.

Surprisingly, many of the plasma cytokine levels remained altered in diagnosed patients compared to the healthy control group even after more than 6 years of having stopped working with ES, the hazard source. Patients with silicosis seem to have a chronic inflammatory process that is consistent with the augmented levels of some pro-inflammatory cytokines, i.e., IL-1β, IL-6, IL-7, IL-8, IL-16, IL-17A, IL-33 and TNF-α were observed in this work.

In vitro experiments have demonstrated that IL-1β and TNF-α secretion by alveolar macrophages is considered an early inflammatory response that leads to progressive tissue damage and fibrosis in coal worker pneumoconiosis (CWP) [25,26]. However, the production of TNF-α and IL-6, but not IL-1β, was observed by human alveolar macrophages exposed to coal dust [27]. Initial TNF-α levels released by stimulated peripheral blood monocytes were related to a progression in CWP even after the end of occupational exposure [28,29]. In human plasma/serum, TNF-α levels in patients with silicosis [16] or IL-1β, IL-6 and TNF-α in CWP patients [30] have also been reported to be increased compared to control groups and even between simple pneumoconiosis and PMF groups, in accordance with the results in the present study. We only observed a significant and progressive increase in TNF-α levels in the HC→SS→PMF groups, suggesting that this factor could play an important role in the development of silicosis. However, contradictory results have been obtained in other studies where only increased plasma IL-6 levels, but not IL-1β, IL-10 and TNF-α levels, were observed [15,31] or in CWP patients in whom the IL-6 level, but not TNF-α, was augmented and correlated with the severity of the disease [32]. Our results showed a clear increase in IL-1β, TNF-α and IL-6 levels in the plasma of patients compared to controls, and for TNF-α, a significant difference was observed between SS and PMF. These results are in line with the results obtained for IL-1β, TNF-α, IL-6 and IL-8 in the bronchoalveolar lavage fluid (BALF) of silicosis patients [33]. On the other hand, no significant difference has been described for these cytokines, IL-1β, TNF-α and IL-6, in patients with natural stone silicosis using the same detection technique [19]. Further studies are necessary to test whether the differences in the levels of those cytokines could be involved in the aggressiveness or rapid progression observed in ES silicosis patients compared to natural stone silicosis patients.

A relevant result was also obtained for IL-8 levels, which were highly increased not only between HCs and patients but also between patients with SS and the group with a more advanced stage of the disease, PMF, which means that this cytokine, along with others, could be a good candidate marker to discriminate between disease stages. This is in line with recently published data where IL-8 levels were associated with progression and death in silicosis [15], with pulmonary impairment in copper smelter workers [34] and with progression in CWP disease [35]; however, in another study with CWP patients, no significant differences were observed in IL-8 levels [36]. 

Other pro-inflammatory cytokines we found to be significantly elevated in the patient groups compared to the control group were IL-7, IL-16, IL-17A and IL-33, with only IL-16 being significantly different in the PMF group compared to any other group. Elevated IL-7 serum levels have been described in patients with natural stone silicosis [19] and sarcoidosis [37] and are associated with the severity of coronavirus disease 19 (COVID-19) [38]. IL-16 acts as a chemoattractant for lymphocytes and monocytes, and their derived macrophages secrete different pro-inflammatory cytokines [39,40]. Although the increase in IL-16 has been related to inflammatory processes such as asthma, colitis, systemic lupus erythematosus and rheumatoid arthritis in silicosis, a decrease in induced sputum has been reported [41]. IL-17 has also been related to inflammation and fibrosis in a silicosis mouse model [42,43]. IL-33 is augmented in plasma patients, and it has been described as promoting the polarization of M1 macrophages to M2 macrophages, producing anti-inflammatory and profibrotic effects [44]. 

With respect to mainly anti-inflammatory cytokines, all of them are elevated in plasma from patients compared to HC. The significant augmentation observed for IL-1RA in the PMF group compared to the SS group and comparing the SS and HC groups is especially remarkable. Thus, this cytokine is a good candidate to be included as a biomarker of disease progression. IL-1RA could be augmented as a mechanism to balance the effect of IL-1β, as has been recently proposed in response to RNA vaccines [45]. We also observed a significant increase in IL-4 and IL-13 levels in plasma in ES silicotic patients, both cytokines are mainly associated with the TH2 response and associated with inflammation and lung fibrosis. Controversial results have reported the role of the TH1/TH2 response in experimental silicosis using animal models [46]. In humans, no clinical trials testing the possible role of IL4 or IL13 in silicosis are known, but some IL4 gene polymorphism studies indicate some susceptibility in CWP [47]. The IL-10 level was also significantly increased in our PMF group compared to the SS or HC groups, in accordance with a previous study [19] but contrary to the results reported in another study with a more limited number of participants comparing silica-exposed versus unexposed subjects [31]. The possible role of this anti-inflammatory cytokine in human silicosis has yet to be reported.

Our data support that some cytokines (IL-4, IL-13 and IL-9) associated with a TH2 response are increased in the plasma of patients with silicosis, while cytokines associated with the TH1 response are practically not altered. Considering that our patients were in a chronic stage for several years, a dominant TH2 response is consistent with the results reported by others [48,49]. IL-18, which can mediate both TH1 and TH2 responses, was significantly augmented in the SS and PMF groups compared to the HC group and in the PMF group compared to the SS group. However, possible changes in INF-γ levels induced by IL-18 were not observed. IL-18 has also been associated with idiopathic pulmonary fibrosis and CWP [50,51].

In mice, it has been demonstrated that IP-10, MCP-1, MIP-1α and MIP-1β, chemokines that could facilitate the trafficking, activation state and recruitment of inflammatory cells into the lungs, are mediated, at least in part, through TNF-α signaling [52]. This is in accordance with the data presented in our work, in which we observed an increase in all chemokine levels in SS and PMF patients compared to the control group, as well as in TNF-α levels (Figure 1). Indeed, the MIP-1α level, as for TNF-α, also exhibited a significant difference between the two silicotic groups, the SS and PMF groups. However, no differences were also reported for TNF-α and MCP-1 levels in plasma in a study including 57 silica-exposed workers (miners and stone carvers) [20]. Previously, MCP-1 has been associated with progression in CWP [53], and an inhibitory role has been assigned to IP-10 in pulmonary fibrosis [54]. The discrepancies observed between the different studies could be attributable to several reasons, such as the different techniques used, representative subject groups, and the nature or composition of the silica particles. All these possible variables will need further study.

TGF-β1 is considered a key fibrogenic cytokine implicated in the manifestation and development of silicosis that can be produced by a plethora of cells in the human lung [55,56]. TGF-β1 levels were significantly increased in SS and PMF patients compared to HCs, but contrary to expectations, no differences were observed between SS and PMF patients. This could indicate that other fibrotic mechanisms could have a role in the progression of lung fibrosis. In this sense, anti-inflammatory cytokines such as IL-4, IL-10 and IL-13 have been proposed to have a profibrotic role in the development of lung fibrosis [57], and precisely, in this work, we have presented data in which the levels of IL-4, IL-10 and IL-13 in plasma are increased in silicotic patients, in line with the TGF-β1 level increase. Because our data were obtained from plasma, we have the limitation of actually knowing the cell sources of TGF-β1 production, but suppressing the general activation and/or expression of TGF-β1 may help us to identify novel and effective therapeutic strategies for silicosis [58,59,60].

VEGF indirectly augments inflammation through the upregulation of cytokine expression (IP-10, MCP-1, IL-8, etc.) and, consequently, chemoattracting inflammatory cells. In addition, although VEGF has been associated with the development of lymphangiogenesis in the early stage of inflammation in silicosis, no significant difference in VEGF-C was observed in the BALF of silicotic patients compared to controls [61]. In human plasma, we also did not observe differences between the HC and SS groups, but we observed a significant difference when comparing the PMF group with either of the aforementioned groups. The VEGF plasma level can be considered another candidate biomarker to discriminate between SS and PMF. Augmenting the expression of soluble VEGF receptor (sVEGFR, acting as a decoy receptor) in mice resulted in an attenuation of pulmonary fibrosis [62], which could be a therapeutic strategy against lung inflammation and pulmonary fibrosis.

Basic fibroblast growth factor (bFGF) has been reported to be produced by mast cells, lung epithelial cells, macrophages and endothelial cells and to be augmented in patients or animal models of silicosis [63,64,65]. However, a previous study using the same technique we used in the present study did not observe any differences in bFGF between a healthy control group and a silicosis group [19]. The explanation of this discrepancy could be due to the number of subjects studied, that they used serum and that we used plasma as a source of metabolites, or just because ES silicosis patients included in our study versus silicosis patients recruited in their work have different behaviors and cytokine production is quite different.

G-CSF and GM-CSF are prototypical granulocyte-mobilizing and granulocyte-macrophage-mobilizing cytokines, respectively. In the present work, as seen by Chen et al. [19], we found that G-CSF levels did show significant differences when comparing the PMF group with the SS or HC groups, but GM-CSF did not show differences at all. In rat models of pulmonary fibrosis, G-CSF treatment exacerbates acute lung injury and pulmonary fibrosis through a mechanism that probably involves the recruitment of neutrophils into the lungs [66,67]. No clinical data on the treatment of silicosis patients with G-CSF have been reported, but its pathway inhibition, as with PDGF, VEGF and FGF signaling inhibition, could attenuate lung fibrosis [68].

This study has some limitations, including the following: (1) It is a cross-sectional study designed to evaluate the cytokine levels in the different studied groups that could be used as a biomarker of ES silicosis disease, and no more conclusions or inferences could be taken without more analysis. (2) Quantitation of environmental respirable silica dust or lung crystal burden in the subjects of the study was not performed, and therefore, we do not know if the response is due to the alveolar silica load or to an individual response or both.

## 4. Materials and Methods

### 4.1. Subjects of the Study

All patients included (*n* = 91) were male workers who were cutting, polishing and finishing engineered stone countertops and were diagnosed with SS (*n* = 53) or with PMF (*n* = 38). They are part of a cohort of patients followed by the Pneumology, Allergy and Thoracic Surgery Department of Puerta del Mar University Hospital in Cádiz (Spain). Patients had been diagnosed with silicosis based upon a history of exposure to silica and chest radiography and/or high-resolution computed tomography (HRCT) and, in some cases, by lung or mediastinal lymph node biopsy. Patients were asked to enroll in the study when they attended a hospital consultation. Respiratory function tests, chest radiographs and HRCT scan classification of these patients have been described previously [13]. The exclusion criteria for a patient from the study were active infection, kidney or liver disease, autoimmune rheumatic disease, or use of immunosuppressive drugs; only oral corticosteroids were accepted at a dose lower than 20 mg per day.

Blood extraction was also performed on 22 healthy control (HC) subjects with no history of exposure to silica dust. All of them were hospital staff workers, and none of them had respiratory symptoms or chronic or acute disease. The medical evaluation before blood sampling was normal in all cases.

### 4.2. Ethics

This study was approved by the institutional Research Ethics Committee of the province of Cadiz (registration n° 90.18, date 29/09/2018). All subjects gave written informed consent following the Declaration of Helsinki. All data were pseudonymized to preclude patient identification and were included in a database to which only the researchers had access.

### 4.3. Plasma Cytokine Analysis

Ten milliliters of venous blood samples were collected into Vacutainer^®^ EDTA tubes (Becton Dickinson, Madrid, Spain). Plasma fractions were obtained after two centrifugations, one at 1500× *g* for 10 min and the second at 2500× *g* for 15 min for the depletion of platelets, and stored at −80 °C until use. Cytokine analysis was performed using (i) Bio-Plex Pro^™^ Human Cytokine 27-plex Assay (Bio-Rad Laboratories Inc., CA, USA) for the analysis of the following cytokines: FGF-basic, Eotaxin, G-CSF, GM-CSF, IFN-γ, IL-1β, IL-1RA, IL-2, IL-4, IL-5, IL-6, IL-7, IL-8, IL-9, IL-10, IL-12 (p70), IL-13, IL-15, IL-17A, IP-10, MCP-1, MIP-1α, MIP-1β, PDGF-BB, RANTES, TNF-α and VEGF, following the instructions of the manufacturer. (ii) Human Luminex Discovery Assay (ref. LXSAHM, R&D Systems) for the analysis of IL-3, IL-16, IL-18, IL-23, IL-33 and CXCL1/GROα, and (iii) a Human TGF-β1 Elisa Kit (ref. RAB0460, from Sigma-Aldrich, Madrid, Spain) as a single ELISA kit for TGF-β1 detection, was used following strictly the instructions of the manufacturer. All determination analyses were performed using Luminex technology FLEXMAP 3D^®^ equipment (Luminex Corporation, TX, USA), but for TGF-β1 colorimetric detection, a BioTek PowerWave HT microplate reader (BioTek Instruments, VT, USA) was used. All samples were measured in duplicate.

### 4.4. Statistics

SPSS software (IBM Statistics) was used for statistical analysis. Initially, the normality distribution of every set of data was established using the Kolmogorov-Smirnov test. Subsequently, one-way ANOVA for multiple (generally three: HC, SS, PMF) groups of data was performed by the ANOVA F test (normal distribution) or by the Kruskal-Wallis test (nonnormal distribution). For comparisons of two groups of data (HC vs. SS, HC vs. PMF or SS vs. PMF), Student’s t-test (for normally distributed data) or the Mann-Whitney U test (for nonnormally distributed data) was used. The chi-square test was used to test relationships between categorical variables. The results are expressed as the mean and standard deviation (SD). A significance level of *p* ≤ 0.05 was adopted for all tests. Excluded data are from those that did not reach minimal detectable values to be included in curve analysis and those considered extreme outliers (3.5 times above or below the mean value).

## 5. Conclusions

To our knowledge, this is the first study that specifically investigates blood cytokines in patients with ES silicosis, in addition to using a broad panel of cytokines. A summary of the results, including altered and unaltered cytokine levels, is shown in Table 2. In particular, the increased levels of IL-1RA, IL-8, IL-10, IL-16, IL-18, TNF-α, MIP-1α, G-CSF and VEGF in PMF patients compared to SS patients could serve as the basis for designing a panel of cytokines, to which others can gradually be added, to serve as a robust biomarker of the severity of ES silicosis. Moreover, recently, some authors have reviewed new information about anti-cytokine therapies and antifibrotic drugs based, mainly, on in vitro studies or animal experimental models [69]. Our study provides relevant information on cytokines involved in the inflammatory process of ES silicosis patients and could be useful to select anti-cytokine agents or antifibrotic drugs with a more suitable profile to slow or stop the progression of the disease.

## Figures and Tables

**Table 1:** Sociodemographic data of participants and pulmonary function values of patients with SS and PMF.

	HC (*n* = 22)	SS (*n* = 53)	PMF (*n* = 38)	*p*
Age *	36.4 ± 8.3	40.1 ± 7.7	41 ± 6.2	0.052 ⁺
Starting Exposure Age *	-	21.2 ± 7.4	21.4 ± 4.3	0.142 ⁺⁺
Duration of Exposure *	-	13.1 ± 6.7	13.3 ± 6.1	0.968 ⁺⁺
Years since cessation of exposure to blood draw *	-	6.4 ± 2.7	7.3 ± 2.5	0.058 ⁺⁺
Smoking status **				0.099 ⁺⁺⁺
Non-Smoker	15 (65.2)	22 (41.5)	15 (39.5)	
Ex-Smoker	5 (21.7)	26 (49.1)	21 (55.3)	
Smoker	3 (13)	5 (9.4)	2 (5.3)	
FEV₁ (mL) *	nd	3386 ± 647	2961 ± 631	0.003
FEV₁ (%) *	nd	87.8 ± 14	76.5 ± 14.8	<0.0001
FVC (mL) *	nd	4341 ± 748	3961 ± 783	0.022
FVC (%) *	nd	90.1 ± 13.3	82.3 ± 14.8	0.01
FEV₁/FVC *	nd	0.77 ± 0.05	0.74 ± 0.07	0.009
DLCO (mmol/min/kPa) *	nd	9.2 ± 1.7	8.3 ± 1.4	0.006
DLCO (%) *	nd	85.4 ± 14.8	77.6 ± 14	0.014

Forced expiratory volume in 1 s (FEV_1_), forced vital capacity (FVC), diffusing capacity of lung for carbon monoxide (DLCO). * Mean ± standard deviation. ** Number of cases (percentage). ⁺ ANOVA F test, ⁺⁺ Mann–Whitney *U* test, ⁺⁺⁺ χ² test; nd, not determined.

**Table 2:** Summary of cytokine levels observed.

Groups Compared	Cytokines Augmented (→)
HC → SS	IL-1β, IL-1RA, IL-4, IL-6, IL-7, IL-8, IL-13, IL-15*, IL-17A, IL-18, IL-33, TNF-α, IP-10, MCP-1, MIP-1α, MIP-1β, TGF-β1, FGF-basic
HC → PMF	IL-1β, IL-1RA, IL-4, IL-6, IL-7, IL-8, IL-9, IL-10, IL-13, IL-15 *, IL-16, IL-18, IL-33, TNF-α, IP-10, MCP-1, MIP-1α, MIP-1β, TGF-β1, FGF-basic, G-CSF, VEGF
SS → PMF	IL-1RA, IL-8, IL-10, IL-16, IL-18, TNF-α, MIP-1α, G-CSF, VEGF
Progressive increase HC → SS → PMF	IL-1RA, IL-8, IL-18, TNF-α, MIP-1α
No significant differences between any of the groups	IL-2, IL-3, IL-5, IL-12 (p70), IL-23, Eotaxin, GM-CSF, IFN-γ, PDGF-BB, RANTES, CXCL1/GROα

* Not detected in HCs but detected in the SS and PMF groups.

## Data Availability

Existing ethical permits do not allow that personal data from this study are deposited in the public domain. The full dataset is available for researchers who meet the criteria for confidential data access as stipulated by participant informed consent and the Institutional Research Ethics Committee of the province of Cadiz (registration n° 90.18, date 29 September 2018), Spain.
